# Supplementary material for: Biopotentials of Collagen Scaffold Impregnated with Plant-Cell-Derived Epidermal Growth Factor in Defective Bone Healing
Source: Materials (Basel). 2023 Apr 24;16(9):3335. doi: 10.3390/ma16093335 (PMC10179640; doi:10.3390/ma16093335)
Supplement: Supplementary file 1 [file materials-16-03335-s001.zip › materials-2330665-supplementary/materials-2330665-supplementary.pdf]

Biopotentials of Collagen Scaffold Impregnated with Plant-Cell-Derived Epidermal Growth Factor in Defective Bone Healing

Sher Bahadur Poudel [1,†], Govinda Bhattarai [2,†], Tae-Ho Kwon [3], Jeong-Chae Lee [2,4,*]

[1] Department of Molecular Pathobiology, New York University College of Dentistry, New York, NY 10010, USA

[2] Cluster for Craniofacial Development & Regeneration Research, Institute of Oral Biosciences, Jeonbuk National University, Jeonju 54896, South Korea

[3] Natural Bio-Materials Inc., Iksan 54631, South Korea

[4] Research Center of Bioactive Materials, Jeonbuk National University, Jeonju 54896, South Korea

Running title: Bioactivities of EGF-loaded collagen scaffold

[*]Correspondence: jeongchae@jbnu.ac.kr; Tel.: +82-63-270-4049; Fax: +82-63-270-4004

[†]These authors equally contributed to this study.

Table S1. Sequences of PCR primers used for real-time PCR

Target	GenBank	Primer	Sequences	Size (bp)
Col1A1	NM_000083.3	F:	AGTGGTTTGGATGGTGCCAA	170
		R:	GCACCATCATTTCCACGAGC	
Runx2	NM_009820.4	F:	GAGGGACTATGGCGTCAAACA	70
		R:	GGATCCCAAAAGAAGCTTTGC	
Osterix	NM_130458.2	F:	TCAGCCGCCCCGATCTTCCA	156
		R:	AATGGGTCCACCGCGCCAAG	
OPN	NM_009263.1	F:	TGGTGGTGATCTAGTGGTGCCAA	148
		R:	CACCGGGAGGGAGGAGGCAA	
OCN	NM_001037939.1	F:	ACTCCGGCGCTACCTTGGGT	109
		R:	CCTGCAGTCTAGCCCTCTGC	
BSP	NM_008318.2	F:	AGACCAGGAGGCGGAGGCAG	123
		R:	TTGGGCAGTTGGAGTGCCGC	
GAPDH	NM_008084.2	F:	GACGGCCGCATCTTCTTGT	65
		R:	CACACCGACCTTCACCATTTT	
